# OCPAT: an online codon-preserved alignment tool for evolutionary genomic analysis of protein coding sequences

**DOI:** 10.1186/1751-0473-2-5

**Published:** 2007-09-18

**Authors:** Guozhen Liu, Monica Uddin, Munirul Islam, Morris Goodman, Lawrence I Grossman, Roberto Romero, Derek E Wildman

**Affiliations:** 1 Center for Molecular Medicine & Genetics, Wayne State University School of Medicine Detroit, MI 48201, USA; 2 Department of Computer Science, Wayne State University, Detroit, MI 48201, USA; 3 Department of Anatomy & Cell Biology Wayne State University School of Medicine Detroit, MI 48201, USA; 4 Perinatology Research Branch, NICHD/NIH/DHHS Bethesda, MD 20892, USA

## Abstract

**Background:**

Rapidly accumulating genome sequence data from multiple species offer powerful opportunities for the detection of DNA sequence evolution. Phylogenetic tree construction and codon-based tests for natural selection are the prevailing tools used to detect functionally important evolutionary change in protein coding sequences. These analyses often require multiple DNA sequence alignments that maintain the correct reading frame for each collection of putative orthologous sequences. Since this feature is not available in most alignment tools, codon reading frames often must be checked manually before evolutionary analyses can commence.

**Results:**

Here we report an online codon-preserved alignment tool (OCPAT) that generates multiple sequence alignments automatically from the coding sequences of any list of human gene IDs and their putative orthologs from genomes of other vertebrate tetrapods. OCPAT is programmed to extract putative orthologous genes from genomes and to align the orthologs with the reading frame maintained in all species. OCPAT also optimizes the alignment by trimming the most variable alignment regions at the 5' and 3' ends of each gene. The resulting output of alignments is returned in several formats, which facilitates further molecular evolutionary analyses by appropriate available software. Alignments are generally robust and reliable, retaining the correct reading frame. The tool can serve as the first step for comparative genomic analyses of protein-coding gene sequences including phylogenetic tree reconstruction and detection of natural selection. We aligned 20,658 human RefSeq mRNAs using OCPAT. Most alignments are missing sequence(s) from at least one species; however, functional annotation clustering of the ~1700 transcripts that were alignable to all species shows that genes involved in multi-subunit protein complexes are highly conserved.

**Conclusion:**

The OCPAT program facilitates large-scale evolutionary and phylogenetic analyses of entire biological processes, pathways, and diseases.

## Background

Multi-species comparisons offer a powerful way to identify functionally important DNA elements that are associated with the evolution of human phenotypes (e.g., the expanded neocortex, language production, and bipedal gait) and diseases that occur mostly in humans (e.g., pre-eclampsia) [1]. Rapidly accumulating whole genome sequence data from vertebrate species provide unprecedented opportunities for evolutionary analyses of protein coding genes. A necessary step in such analyses is the construction of in-frame multiple sequence alignments. Commonly used alignment tools such as CLUSTAL and T-COFFEE [2,3] do not retain reading frame information, thus the achievement of in-frame alignments usually requires manual curation, which is impractical at a genome-wide scale. Moreover, genomic tools such as threaded blockset aligners [4] derive the reading frame from a single species and allow the others to have frame-shifts, which can affect downstream calculations of DNA substitution rates that are based on codon models. Therefore, none of these tools is wholly appropriate for phylogenetic analyses based on protein-coding models of sequence evolution [5]. To infer non-synonymous and synonymous substitutions for a large gene set, tools that automate codon-preserved alignments are required.

To address these issues, we developed a tool to automate gene alignments on a genome-wide scale with the reading-frame preserved for each set of putatively orthologous coding sequences. The tool is called OCPAT (Online Codon-Preserved Alignment Tool).

## Implementation

The OCPAT pipeline is composed of 1) a user interface [6], 2) a CGI program to handle queries, 3) a genomic database to store sequences, and 4) the main program to generate alignments. Output is stored on a server and users are notified through email of URLs containing their results.

The current version of OCPAT aligns genes from *Homo sapiens *(human) [7], *Pan troglodytes *(chimpanzee) [8], *Macaca mulatta *(Rhesus macaque) [9], *Mus musculus *(mouse) [10], *Rattus norvegicus *(rat) [11], *Oryctolagus cuniculus *(rabbit), *Canis familiaris *(dog) [12], *Bos taurus *(cow), *Dasypus novemcinctus *(armadillo), *Loxodonta africana *(elephant), *Echinops telfairi *(tenrec), *Monodelphis domestica *(opossum) [13], *Ornithorhynchus anatinus *(platypus), *Gallus gallus *(chicken) [14], and *Xenopus tropicalis *(frog). mRNA and/or cDNA files are downloaded from the RefSeq mRNA databases [15], the ENSEMBL cDNA databases [16], and the NR (Non-redundant) mRNAs [17]. mRNA/cDNA sequences are then sorted by species and formatted and indexed using the "formatdb" program [18]. The GenBank formatted human mRNA and protein sequences are downloaded from RefSeq as well [19]. For the analysis described, data were updated on November 2, 2006.

**Figure 1:**
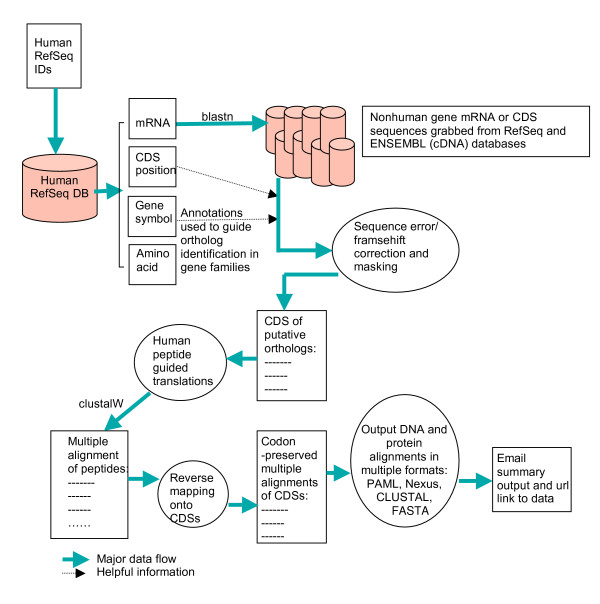
**Workflow of OCPAT**. The pipeline implements the steps as shown in the figure. The human RefSeq mRNA serves as the initial query by default. Putative orthologs are defined by sequence similarity and gene symbol. Frameshift causing substitutions are masked, and OCPAT does not distinguish true frameshifts from sequencing errors. Unlike other alignment tools, a RefSeq golden peptide sequence record guides OCPAT alignments rather than the predicted amino acid sequence derived from the mRNA record.

The procedure for obtaining and aligning sequences is executed using multiple available tools linked to one another through perl modules and scripts [see additional file 1]. OCPAT implements the following steps (Fig. 1):

1. **Submission**: Submits a list of human RefSeq IDs (e.g., NM_002425, NM_181814) in single-column format. Users can choose which (or all) of 14 additional taxa will be included in alignments.

2. **Ortholog extraction: **Retrieves mRNAs or CDs from the other species by BLAST [20] search of respective cDNA or mRNA databases using the human CDs as the query. In the case of multiple sequences from one organism showing high similarity to the human CDs (i.e. less than 5% difference between paralogs in the nonhuman organism), the annotations (e.g. gene symbol) of those genes are used to choose the putative ortholog. OCPAT defines putative orthologs solely by sequence similarity. OCPAT also measures sequence concordance, which is a measure of the relative proportions of subject to query alignment length. Sequence concordance is calculated where Concordance = 2 * matched sequence length/(query sequence length + aligned subject sequence length). Aligned sequences shorter than half the length of the queried human sequence are eliminated from further consideration.

3. **Error correction: **Pre-aligns sequences using CLUSTALW [3]. Searches these alignments for possible places where only one or two sequences has a frame shift introduced by nucleotide insertions or deletions while all the other sequences are perfectly aligned to each other. Indels causing gaps in these multiple sequence alignments are filled with "N"s so the subsequent translation does not cause frame shifting.

4. **Determination of reading frame**: Obtains a human peptide for the queried RefSeq directly from the human.rna.gbff file. Translates putative orthologous gene sequences into peptides and determines the reading frame of each gene by aligning the translated sequence to the human peptide using the bl2seq program [21]. An "X" is used for amino acids derived from codons containing ambiguous nucleotides. Each sequence is then trimmed so codons correctly begin with the first nucleotide position. Peptides for the orthologous gene sequences are aligned using the CLUSTALW program [3]. Aligned peptides are then "translated" back to their corresponding cDNA sequences by sequence mapping in which the "reverse translation" is directed by correlated CDS and peptide positions (e.g., the Nth amino acid in the peptide maps back to the 3N-2, 3N-1, 3N nucleotides in the CDS), thus avoiding the problem of codon degeneracy. This translation, alignment and "reverse translation" procedure generates alignments that preserve the codon reading frames.

5. **Core alignment**: Evaluates the cDNA alignment for the core alignment region, in which the suboptimal alignments at the beginning and end of genes (often due to poor predictions or sequence errors) are removed. A sliding window of three consecutive amino acids, beginning from the 5' end, is moved across the multiple sequence alignment. The "identical count" is determined by calculating the number of identical amino acids at each position in a three-amino-acid window. For a multiple sequence alignment of N sequences, the maximum "identical count" per window is 3N. When the "identical count" reaches 2.2N, the first amino acid in the sliding window is marked as the start point of the core alignment. This represents slightly over 70% identity in the alignments. The same sliding window strategy trims the 3' end of the core alignment. Large, single species insertions are also removed from the core alignments. The remaining "core alignments" always begin with the first nucleotide within a codon and end at the third nucleotide within a codon

6. **Output: **Produces NEXUS-, PHYLIP-, and CLUSTAL- formatted files, which can be utilized by a variety of phylogenetic programs including PAUP*, MacClade, PAML, and Mr.Bayes [5,22-24]. Additional output files include standard error files and a summary file (ocpat.align.sum).

## Results

Using OCPAT we generated 20,658 multiple sequence alignments derived from human mRNA RefSeq IDs. Among these alignments 10,258 included 10 or more species. The pairwise numbers of alignable putative orthologous sequences is shown in Table 1, and a recent version of alignment files for putative orthologous sequences are available at [25]. All putative orthologs are considered provisional, and certainly there are some non-orthologous sequences included in individual gene alignments due to genome assembly errors, lineage specific gene duplications, and ascertainment errors. As expected, we obtained a greater number of putative human orthologs from species more closely related to human (e.g., chimpanzee, macaque) than from more distantly related species (e.g., chicken, frog). We also found that mammal species whose genomes were sequenced at 2-fold coverage had fewer recovered orthologs than did mammals with higher quality sequences. Despite these limitations, there are 1,698 human RefSeqs for which we were able to obtain putative orthologs from all taxa queried (N = 13; the platypus Genebuild was not available as of Nov. 2, 2006). Phylogenetic analyses have been conducted on these genes using parsimony, distance, likelihood, and Bayesian methods [26].

**Table 1:** Pairwise taxon by putative ortholog matrix among 14 species and 20658 RefSeq mRNA Gene IDs

	**Human**	**Chimp**	**Macaque**	**Mouse**	**Rat**	**Rabbit**	**Dog**	**Cow**	**Armadillo**	**Elephant**	**Tenrec**	**Opossum**	**Chicken**	**Frog**
**Human**	-	19798	20078	18009	17732	10942	18964	18437	8924	9987	10509	13250	9126	4931
**Chimp**	860	-	19574	17561	17336	11390	18550	18093	9536	10551	10999	13314	9540	5617
**Macaque**	580	1084	-	17825	17588	11254	18740	18299	9322	10323	10811	13396	9444	5421
**Mouse**	2649	3097	2833	-	19779	12243	18385	18222	10429	11350	12464	15479	11651	7552
**Rat**	2926	3322	3070	879	-	12294	18226	18093	10540	11481	12579	15554	11812	7811
**Rabbit**	9716	9268	9404	8415	8364	-	11844	12159	13196	13153	13417	12582	11936	11405
**Dog**	1694	2108	1918	2273	2432	8814	-	18703	10030	11047	11729	14430	10564	6549
**Cow**	2221	2565	2359	2436	2565	8499	1955	-	10411	11380	12022	14353	10639	6906
**Armadillo**	11734	11122	11336	10229	10118	7462	10628	10247	-	7329	13281	11718	12034	12437
**Elephant**	10671	10107	10335	9308	9177	7505	9611	9278	7329	-	13334	11965	11811	11838
**Tenrec**	10149	9659	9847	8194	8079	7241	8929	8636	7377	7324	-	13265	12741	12022
**Opossum**	7408	7344	7262	5179	5104	8076	6228	6305	8940	8693	7393	-	15290	11921
**Chicken**	11532	11118	11214	9007	8846	8722	10094	10019	8624	8847	7917	5368	-	15769
**Frog**	15727	15041	15237	13106	12847	9253	14109	13752	8221	8820	8636	8737	4889	-

above diagonal = shared RefSeqs

below diagonal = not shared RefSeqs

To explore the biological significance of the genes found in all species we conducted a functional annotation clustering analysis using the default settings of the DAVID package [27]. The results of this analysis indicated a statistically significant over-representation of genes that encode proteins found in multi-subunit complexes (n = 263 RefSeqs; p = 5.0E-39). Other overrepresented annotations in functional clusters include one comprised of ribosomal proteins and one containing proteins in the histone core. We consider the genes with putative orthologs for all species to be a good indicator of conservation (i.e., more identifiable orthologs indicates more functional constraint on the protein). Taken together, these results suggest that protein-protein interactions in multi-subunit complexes are under considerable evolutionary constraint. Therefore, mutations in these proteins are possibly more likely to be harmful when they occur.

## Discussion

In silico gene prediction algorithms often fail at the 5' and 3' ends of a gene. Consequently, the 5'end and the 3'end of the predicted ORFs are error prone. This can lead to low-quality alignments in the 5' ends and the 3' ends of a given gene. OCPAT trims the low-quality alignment regions at the ends. The remaining high-quality core alignments in the middle of the gene may be less "noisy" than whole alignments. We also found that many of the genes predicted for opossum, chicken, and frog have large insertions when compared to genes from placental mammals. Therefore, if only one species has a big insertion while all the others do not, OCPAT removes the insertion. This treatment is most effective when there are other sequences that partially overlap the insertion. By removing these large insertions, the smaller overlapping regions are not "lost" as alignment gaps in subsequent phylogenetic analyses. If, after the initial run, the user finds the inclusion of one species disrupts the alignment due to factors such as poor gene prediction and short length, the user can re-run OCPAT for that gene and eliminate any disrupting sequences.

## Conclusion

In summary, we provide a simple tool for aligning genes with the protein coding frames preserved. Alignments are formatted so they can be applied to evolutionary analyses using appropriate software. The tool is effective for creating alignments on a genome-wide scale. Future versions of OCPAT will use the genome sequences of additional species.

## Availability and requirements

Project name: OCPAT

Project home page: .

Operating system(s): Mac OS X or Solaris 9/10; web server version is platform independent

Programming language: Perl

Other requirements: for the command line; NCBI BLAST utility, CLUSTAL; genome data

License: GNU General Public License

Any restrictions to use by non-academics: None

## Abbreviations

OCPAT - Online Codon Preserved Alignment Tool.

## Competing interests

The author(s) declare that they have no competing interests.

## Authors' contributions

All authors have read and approved the final manuscript. DEW defined the problem and designed the project. GL and MI wrote the code and implemented OCPAT. MU, GL, MI, and DEW tested and debugged the programs. All authors participated in the manuscript preparation.

## Supplementary Material

Additional file 1 Ocpat.pl. OCPAT Source Code (perl script) Click here for file
